# A case of radiation-induced sternal malignant fibrous histiocytoma treated with neoadjuvant chemotherapy and surgical resection

**DOI:** 10.1186/1477-7819-6-138

**Published:** 2008-12-30

**Authors:** Bulent Kocer, Gultekin Gulbahar, Bulent Erdogan, Burcin Budakoglu, Selim Erekul, Koray Dural, Unal Sakinci

**Affiliations:** 1 Numune Education and Research Hospital, Thoracic Surgery Department, Ankara, Turkey; 2 Numune Education and Research Hospital, Plastic and Reconstructive Surgery Department, Ankara, Turkey; 3 Numune Education and Research Hospital, Medical Oncology Department, Ankara, Turkey; 4 Ankara University School of Medicine, Pathology Department, Ankara, Turkey

## Abstract

**Background:**

Primary sternal malignant fibrous histiyocytoma (MFH) is highly rare. Effective treatment modality is surgical resection with wide margins. However, to date, the effects of radiotherapy or chemotherapy has not been clearly defined.

**Case presentation:**

Herein, we aimed to present a 50-year old female patient with MFH occurred in the radiotherapy field who had had surgical procedure for breast cancer 19 years ago and had followed by radiotherapy. Neoadjuvant chemotherapy was applied for MFH due to cardiac and mediastinal vascular invasion. Wide resection was carried out for the mass after having been decreased in size following neoadjuvant chemotherapy.

**Conclusion:**

Neoadjuvant chemotherapy was an effective method. In planning the surgical resection, the size of the tumor before chemotherapy should be considered as the initial size and surgical margins should be determined accordingly.

## Background

Primary sternum tumors are rare entities, almost all of which have a malignant progression [1]. Chondrosarcoma is the most common primary sternum tumor[2]. MFH usually results from radiation scar [3]. Effective treatment modality is surgical resection with wide margins. Radiation-induced MFH is rare and its treatment regards experience.

## Case presentation

**Figure 1:**
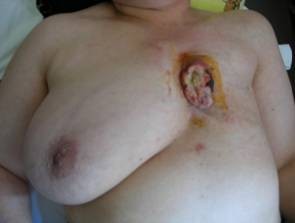
**The ulcerated mass originating from the sternum**.

**Figure 2:**
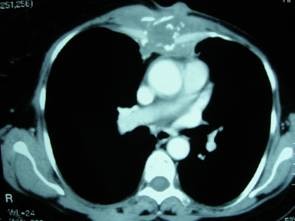
**The chest CT image of the patient**.

A 50-year-old female patient applied with ulcerated and painful lesion on the sternum (Figure 1). The patient had the complaints for 3 months, during which the lesion gradually grew. The personal history of the patient revealed that she had undergone total modified radical mastectomy on the left and axillary lymph node dissection for infiltrative ductal breast carcinoma 19 years before and received radiotherapy and adjuvant chemotherapy. She had also received hormonotherapy for 2 years. In the physical examination, an ulcerated lesion of 5 × 6 cm in diameter, which was fixed on the sternum and rising above the skin surface, was palpated. In the laboratory evaluations, the serum gama-glutamyl transpherase (GGT) level was 194 U/L and the other biochemical analyses and hematological parameters including the tumor markers were normal. The lateral x-rays showed destruction of the sternum. Thorax computerized tomography (CT) and magnetic resonance imaging evaluations indicated a lesion of 5 × 6 cm in size that had caused nodularity on the skin and marked destruction in the sternum (Figure 2). The diagnosis based on the incisional biopsy was MFH.

The patient did not have any findings of distant metastasis, but was considered inoperable because of local invasion. A chemotherapy protocol of iphosphamid 2500 mg/m^2 ^(3 days), mesna 2500 mg/m^2 ^(3 days), and adriablastina 50 mg/m^2 ^(1 day) (IMA) was started with 21-day intervals. After two cures of chemotherapy, the size of the lesion was 3 × 3 cm. Repeated thorax CT showed regression of the local invasion, and the tumor was operable. Thus, operation was planned.

### Treatment

Intraoperatively, the lesion on the corpus stern was totally excised with a 4-cm margin along with the health tissue. The manubrium sterni was preserved. To provide stabilization, prolene mesh and methyl methacrylate were used. Then, the areolar fatty tissue and skin composite transposition flap was formed and transpositioned on the prolene mesh. A drain with negative pressure was placed under the transposition flap. The remaining right breast tissue was advanced along the inframammarian sulcus and the flap donor area was closed.

### Pathological findings

**Figure 3:**
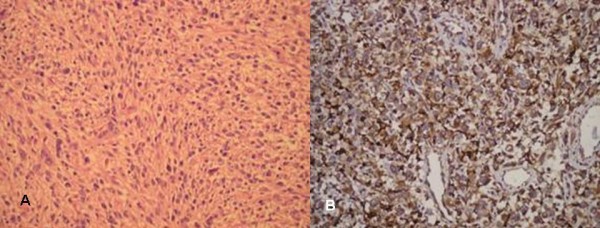
**A) The tumor consisted of cells with spindle character forming non-marked whirlpool-like structures at certain places and fewer oval cells (H&E.200×)**. B) It has been observed that tumor cells were strongly stained with CD68 immunohistochemically (CD68 200×).

The histopathological evaluation of the incisional biopsy material showed a tumor growth of mesenchymal character with cellular appearance. The tumor consisted of cells with spindle character forming non-marked storiform pattern at certain places and fewer polygonal cells. There were marked nuclear pleomorphism and common mitosis. Atypical mitoses and tumor giant cells with bizarre nuclei were not infrequent. Necrotic areas were present. With these properties the tumor was considered as high grade malignant pleomorphic mesenchymal tumor. Immunohistochemically, the tumor cells showed strong cytoplasmic staining with CD68, a histiocyt marker. Therefore, histopathological diagnosis was MFH. (Figures 3 a-b)

### Postoperative follow-up

In the early postoperative period, recurrence was observed. The patient was applied adjuvant chemotherapy. In the postoperative 11^th ^month, cranial metastasis was detected and the patient died in the postoperative 13^th ^month.

## Discussion

Tumors with a sternum origin are a very rare type of primary thoracic wall tumor. Tumors of the sternum usually have a malignant nature. The most common histopathological diagnosis is chondrosarcoma [2]. Although MFH is the most common soft tissue sarcoma, it is rarely located on the thoracic wall and particularly on the sternum[4,5]. In a retrospective study of 64 years, nearly half of the 54 patients with primary sternum tumor were diagnosed with chondrosarcoma, while only one of these patients (1.8%) was diagnosed with MFH [2]. MFH has been reported to be more common among patients who have received radiotherapy [3].

Ionized radiation is clearly known to trigger sarcoma formation [6]. Cahan *et al *in 1948 defined the criteria for post-radiation bone sarcoma. The requirements are: (a) evidence of an initial distinct malignant tumor different from the subsequent sarcoma, (b) development of the second malignant tumor in an irradiated field, (c) long interval between radiation and development of sarcoma and (d) histological confirmation of sarcoma [7]. This case report fulfils these criteria.

In establishing histopathological diagnosis, percutaneous fine needle biopsy is preferred because it is easily applied and has low risk of complications. However, variable morphological characteristics of MFH may render diagnosis challenging [5]. Nonaka *et al *have presented a similar patient in whom the preoperative diagnosis could not be established through percutaneous needle biopsy and due to the destruction in the sternum, complete surgical resection was preferred in the diagnosis and treatment of the patient [8]. Nevertheless, preoperative histopathological diagnosis may lead to considerations for different treatment alternatives such as neoadjuvant chemotherapy in determining a treatment strategy. Unlike percutaneous needle biopsy, incisional biopsy may provide sufficient amount of tissue that will facilitate immune staining. Accordingly, we preferred incisional biopsy in establishing preoperative diagnosis. Neoadjuvant chemotherapy is applicable particularly in case of lesions with mediastinal invasion or lesions of extremely large sizes. In our patient, the size of the lesion was 50% smaller and the findings of mediastinal invasion regressed upon two cures of neoadjuvant chemotherapy. This shows that preoperative neoadjuvant chemotherapy is an effective method.

Following the diagnosis of malignant primary chest wall tumors, surgical resection of a wide area is required. Despite various views on the extent of resection area, many earlier studies have recommended en bloc resection of the tumor, covering an area of 4 cm from all the sides along with the structures such as the lungs, thymus, and pericardial tissues that may be invaded by the tumor [9,10]. By reconstruction of the resultant defect, total closure of the defect and providing structural stability should be aimed. Any defects of smaller than 5 cm at any location on the thoracic wall and any defects smaller than 10 cm on the posterior wall of the thorax can be closed primarily and usually do not require reconstruction. However, reconstruction of any larger defects is essential. In achieving structural stability, methyl methacrylate has been recommended in addition to Marlex, Mersilene, or Prolene Mesh, while in covering the defect, the most commonly used method has been pectoralis major or latissimus dorsi musculocutaneous flap transposition depending on the location of the defect [10]. The lesion of the patient was removed en bloc. In covering the defect, pectoralis major, and in providing the stability of the chest wall, Prolene Mesh™ (Ethicon, Inc., Somerville, NJ) and methyl methacrylate were used.

The recurrence in the postoperative any follow-up period delineates the need for a sufficient resection margin all around the lesion during the surgery. Neoadjuvant chemotherapy can decrease the unnecessary resection around the mass as the margins of which can be shrunken after the neoadjuvant chemotherapy. In our patient, the diameter of the tumor decreased by 3 cm and findings of invasion to the mediastinal structures disappeared after neoadjuvant chemotherapy. It seemed that en bloc resection of the tumor with a vital tissue margin of 4 cm was not totally sufficient in our case due to recurrence after surgery although the size of the tumor had gotten smaller after neoadjuvant chemotherapy. Perhaps, the preoperative size of the tumor should have been considered as the basal measurement, and the decision for the resection extent should be marked as 4 cm vital tissue encircling the original mass before chemotherapy. Taking this into consideration, resection margins should have been like 7 cm of vital tissue from the center of the original mass in our case.

## Conclusion

Neoadjuvant chemotherapy was an effective method in this related case, and we recommend its use in such cases. In planning the surgical resection of the tumor after neoadjuvant chemotherapy, when possible, the size of the tumor before chemotherapy should be considered as the initial size and surgical margins should be determined accordingly. Complete surgical resection is the desired outcome after neoadjuvant chemotherapy which might provide downsizing as happened in our case saving vital tissues to be resected. En bloc resection of the tumor occupying an area of 4 cm vital tissue apart from all along the wound margins with the structures such as the lungs, thymus, and pericardial tissues that may be invaded by the tumor. Afterwards, total closure of the defect by reconstruction using synthetic materials like meshes should be provided to protect the structural stability.

To sum up, it seems neoadjuvant chemotherapy is an effective method in MFH cases before performing surgical resection. Besides, we believe that further studies about its indication are still being warranted in large series in this field.

## Abbreviations

MFH: Malignant fibrous histiyocytoma; CT: Computerized Tomography.

## Consent

Written informed consent was obtained from the patient for publication of this case report and any accompanying images. A copy of the written consent is available for review by the Editor-in-Chief of this journal.

## Competing interests

The authors declare that they have no competing interests.

## Authors' contributions

BK was involved in study conception and design and reviewing previous research. GG was involved in scquisition of data and drafting of manuscript. BE was involved in study conception and design. BB was involved in study conception and design. SE was involved in study conception and design and preparing photographs. KD was involved in analysis and interpretation of data. US was involved in critical revision and project coordination.
